# Parenthood, Economics, and Knowledge

**Published:** 1920-05-01

**Authors:** 


THE PUBLIC WELL-BEING—(*continued*)

### Parenthood, Economics, and Knowledge.

### A Wide Problem.

The inseparable relations between economics and the race question, and, in fact, between economics and many leading subjects of public welfare, are becoming more clearly marked than ever. The increase in the cost of living for babies, for instance, is between 300 and 400 per cent. since the war, and it was wittily said the other day that if the children under two had a strong representative organisation there would be great to-do about the Board of Trade figures on prices. The housing problem, which is now largely an economic question, is responsible for much squalor, ill-health, and immorality. The unhealthy conditions in various industries is partly an economic and partly an industrial question, and it is certain that the students of public health, and the directors of public health organisation must also be students of all the conditions of social life in its various aspects if their complicated problems are to be satisfactorily settled. It requires a broad view and broad knowledge, and then a dissemination of the conclusions reached. Too great an emphasis on this last point can never be made. It is notorious that many of the best-paid workers never use their money to improve their conditions. While there are many who, of course, do so, can it be denied, for example, that there are numbers who never devote their added prosperity to finding better housing accommodation? The facile answer to that at this moment is that such accommodation is not to be had, but it is a fact that before the war this reluctance was manifest. How many devote added prosperity to purer food supplies? Tinned foods and all the rest of it are easy to get; they save the harassed and often overworked housewife considerable trouble, and what has been good enough in the past is considered good enough to go on with; often, no doubt, the old diet is preferred as a matter of choice. There are certainly signs that those who have had little chance hitherto are genuinely anxious to share the better things of life in reality, but it is still hard to believe that the great movement to-day for a higher standard of living finds its impetus, as we are invariably told it does, in the spiritual desire for the finer shades of physical and mental enjoyment. So far as the contention of the leaders of the movement is true, so far will its benefits be national. It will be seen in better education, which can only follow a greater love of education. It will be found in a far wider conception of the inestimable boon of individual regard for health, and it will be accompanied by a richer tone in national life. It would be optimistic to say there are obvious signs now of far-reaching evolution in that direction.

#### The Race Question.

To return to the race question: there have been some remarkable facts brought to the attention of the public within the past week. Dr. Stevenson, the superintendent of statistics, in a lecture on the fertility of various social classes, said that in the deficient fertility of the classes which, having achieved most success in life, were presumably best endowed with the qualifications for its achievement, the nation was confronted with a new and formidable fact—the professions, which formed the purest examples of middle-class occupations, were exceedingly infertile. The total fertility of all the professions tabulated, except Nonconformist ministers, was underneath the lowest standard, though their very small rate of child mortality caused the clergy of the Established Church slightly to exceed that standard in regard to effective fertility. The exceptionally-low figure for naval and military officers might be due to circumstances in their case rendering the maintenance of a family specially difficult, but the failure of this fine stock to reproduce itself was none the less to be regretted.

The most remarkable instance of all was that of persons describing themselves as of "private means." In their case, presumably, the anxieties and difficulties which militated against fertility were at a minimum, but their fertility was also at a minimum. The reason might conceivably be that the more energetic and capable of the class referred to did follow some definite occupation, and that their fertility was higher than that of the inferior remainder of their class.

The effect of female occupation in lowering fertility was clearly established. If we attributed to human volition the fall which had occurred in the nation's fertility, the facts to which he had referred were readily explained, but if we refused to acknowledge this agency it was necessary to assume the reduction of female fertility by non-domestic work as a law of nature.

#### Relative Poverty.

These are serious conclusions which must be borne in mind in the consideration of the trend of social conditions of to-day. It is very difficult to say definitely which of Dr. Stevenson's explanations is nearer the mark. On the face of it, it does appear that the greater the share that women play in the professions and, still more, in public work, the less opportunity have they of becoming mothers of any but quite small families, though there are examples of fine-spirited public women of the last twenty years—to go back no further—who have been equally fine mothers; it may well be, too, that many women who enter public life are those who, in any circumstances, have a need of finding spiritual outlet in other ways than by motherhood, and in preference to motherhood. It can also scarcely be denied that there exists to-day a far greater degree of deliberate limitation of parenthood, often on healthy principles (not, however, beyond controversy), often, on debased feelings. Another factor to be considered in dealing as between professional classes, "private means" classes, and other classes, is that variations in wealth are not only actual but relative. A family whose income of £1,000 is reduced to £600 is poorer, relatively, than a family whose income of £400 has risen to £600, for the standard of life, education of children, and so on, are regarded from different angles. The probability is that the influences of to-day will still further emphasise the tendencies which Dr. Stevenson outlined, and one obvious duty in meeting this position is to raise the general level of culture and of the sense of responsibility. Education is the great primary remedy for half our ills.

